# Th2 skewing in patients with disseminated coccidioidomycosis

**DOI:** 10.1172/jci.insight.199941

**Published:** 2026-04-21

**Authors:** Timothy J. Thauland, Smriti S. Nagarajan, Alexis V. Stephens, Samantha L. Jensen, Anviksha Srivastava, Miguel A. Moreno Lastre, Terrie S. Ahn, Chantana Bun, Michael T. Trump, Royce H. Johnson, George R. Thompson, Maria I. Garcia-Lloret, Valerie A. Arboleda, Manish J. Butte

**Affiliations:** 1 Department of Pediatrics, Division of Immunology, Allergy, and Rheumatology, and; 2 Department of Human Genetics, UCLA, Los Angeles, California, USA.; 3 Department of Medicine, Division of Infectious Diseases, Kern Medical Center, Bakersfield, California, USA.; 4 Department of Medicine, Division of Infectious Diseases, UCLA, Los Angeles, California, USA.; 5 Valley Fever Institute at Kern Medical, Bakersfield, California, USA.; 6 Department of Medicine, Division of Infectious Diseases, and; 7 Department of Medical Microbiology and Immunology, University of California, Davis, Sacramento, California, USA.; 8 Department of Microbiology, Immunology, and Molecular Genetics, UCLA, Los Angeles, California, USA.

**Keywords:** Immunology, Infectious disease, Adaptive immunity, Fungal infections, Immunotherapy

## Abstract

**BACKGROUND:**

Disseminated coccidioidomycosis (DCM) is an often fatal and otherwise intractable condition requiring lifelong antifungal treatment. We have previously shown that a deranged polarization of CD4^+^ T cells toward a Th2 phenotype can exist in the context of DCM. Here, we studied a large population to determine the frequency of abnormal Th2 skewing of CD4^+^ T cells in patients with coccidioidomycosis and to identify underlying genetic mechanisms supporting this phenotype.

**METHODS:**

We collected PBMCs from 204 patients with coccidioidomycosis, including 96 patients with disseminated disease. We measured immune phenotypes and cytokine production by CD4^+^ T cells from patients and healthy controls, and comparisons between groups were made based on disease severity and demographics. Whole-genome sequencing was conducted on 180 individuals who also had cytokine profiling.

**RESULTS:**

We found that approximately 25% of patients with DCM had a CD4^+^ T cell compartment that was abnormally skewed toward a Th2 phenotype, and Th2 skewing was highly correlated with male sex. Coculture of T cells with the IL-4R/IL-13R–blocking antibody dupilumab reduced Th2 skewing. Sequencing revealed rare variants in genes involved in the IL-12/IFN-γ axis in several Th2-skewed patients, and we validated one such variant in *IFNGR1* as hypomorphic.

**CONCLUSION:**

Patients with DCM, especially males, should be screened for Th2 skewing of CD4^+^ T cells. Patients with Th2 skewing should be additionally screened for genetic defects in the IL-12/IFN-γ axis. Our findings give a mechanistic rationale for blockade of IL-4R in Th2-skewed patients with refractory coccidioidomycosis.

**FUNDING:**

National Institute of Allergy and Infectious Diseases/NIH grants R21 AI149654 and U19 AI166059 and University of California Office of the President grant VFR-19-633386.

## Abstract 2

Severe Valley fever in males often features heightened allergy-like immunity, predisposed in some by genetic mutations, which suggests that targeted immune treatments can strengthen defenses.

## Introduction

Coccidioides fungi (*Coccidioides immitis* and *Coccidioides posadasii*) are endemic to arid regions of the American southwest and are responsible for a substantial disease burden. There are approximately 200,000–350,000 infections per year in the United States, with 20,000 cases requiring medical attention and reaching clinical diagnosis (1). Approximately 60% of infections are asymptomatic, while in 40% of cases, coccidioidomycosis causes a relatively mild, self-limited respiratory disease called “Valley fever” (2). Approximately 1% of *Coccidioides* infections escape the lungs, resulting in disseminated coccidioidomycosis (DCM). DCM can manifest in the skin, bones, and nervous system causing marked morbidity and a mortality rate of 30% in the first 5 years after dissemination (3). If they survive, patients with DCM typically require antifungal treatment for life.

Known risk factors for progressing to DCM include age, sex, pregnancy, and race (4). Several monogenic variants in host-defense genes have been shown to predispose for DCM, including in the pathways recognizing β-glucan in the fungal cell wall or producing hydrogen peroxide in response to infection (5). Furthermore, patients with inborn defects in the IL-12/IFN-γ axis are at higher risk of developing DCM upon infection (6–10). At present, there is no known laboratory test or clinical algorithm available to predict whether infection with *Coccidioides* will remain as uncomplicated Valley fever or progress to dissemination.

Protective immunity to coccidioidomycosis requires a robust Type-1 response, characterized by the production of IFN-γ by *Coccidioides*-specific CD4^+^ Th1 cells (11). On the other hand, Type-2 immunity, characterized by the production of IL-4, IL-5, and IL-13, confers susceptibility to invasive fungal infections by impairing innate immune defenses (12–14). *Coccidioides* vaccines in development require IFN-γ production for protection (15–17), and recombinant IFN-γ has been successfully used to treat severe and refractory cases of coccidioidomycosis (18). Mechanistically, lung immune responses in mouse models that distinguish resistant strains (DBA/2) from susceptible ones (C57BL/6) show that increased IFN-γ production correlates with resistance while increased IL-4 production correlates with susceptibility (19). Inducing Type-2 responses in the lung may be a form of immune evasion for certain fungi, driven by chitins in the fungal cell wall that elicit epithelial defenses, including Type-2 alarmins, among other factors (12). In humans, elevations of IgE and eosinophilia have been associated with more severe coccidioidomycosis (20–22). Additionally, Stat3-mutated hyper-IgE (Job) syndrome, a disorder that skews T cells toward Type-2 immunity and is characterized by a defective Th17 response, has been reported in patients with severe coccidioidomycosis (23, 24).

The Pirofski-Casadevall damage-response framework suggests that therapeutic dampening of dysregulated inflammatory responses can be beneficial to the host (25). Indeed, targeted immunosuppression can overcome the detrimental effects of Type-2 immunity, as antibody blockade of IL-4 in mouse models reduces fungal burden (26). We previously reported a case of life-threatening DCM characterized by a defect in Type-1 immunity (27). We found that the patient’s CD4^+^ T cells showed a strong Th2 phenotype, with a large majority of cytokine-producing T cells making IL-4 but not IFN-γ. The patient was successfully treated with a combination of recombinant IFN-γ (augmenting Th1 responses) and an anti–IL-4R monoclonal antibody (dupilumab, mitigating Th2 responses). These treatments markedly improved the patient’s lesions concomitant with an increase in IFN-γ–producing Th1 cells and a decrease in IL-4–producing Th2 cells (27). We recently published a case series of another 14 patients treated with the anti–IL-4R monoclonal antibody (11 of whom also received IFN-γ), showing both a reduction in the Th2/Th1 ratio and clinical improvement (28). This case offered hope that a subset of patients with severe coccidioidomycosis could benefit from an immunomodulatory strategy.

In most healthy people, CD4^+^ T cells that are restimulated ex vivo are skewed toward a Th1 phenotype (29). Our success in treating a DCM patient with Th2 skewing coupled with the knowledge that Type-1 immunity is essential for a successful immune response to coccidioides infection led us to ask how prevalent deranged Th2 skewing of CD4^+^ T cells is in patients with coccidioidomycosis.

## Results

**Figure 1:**
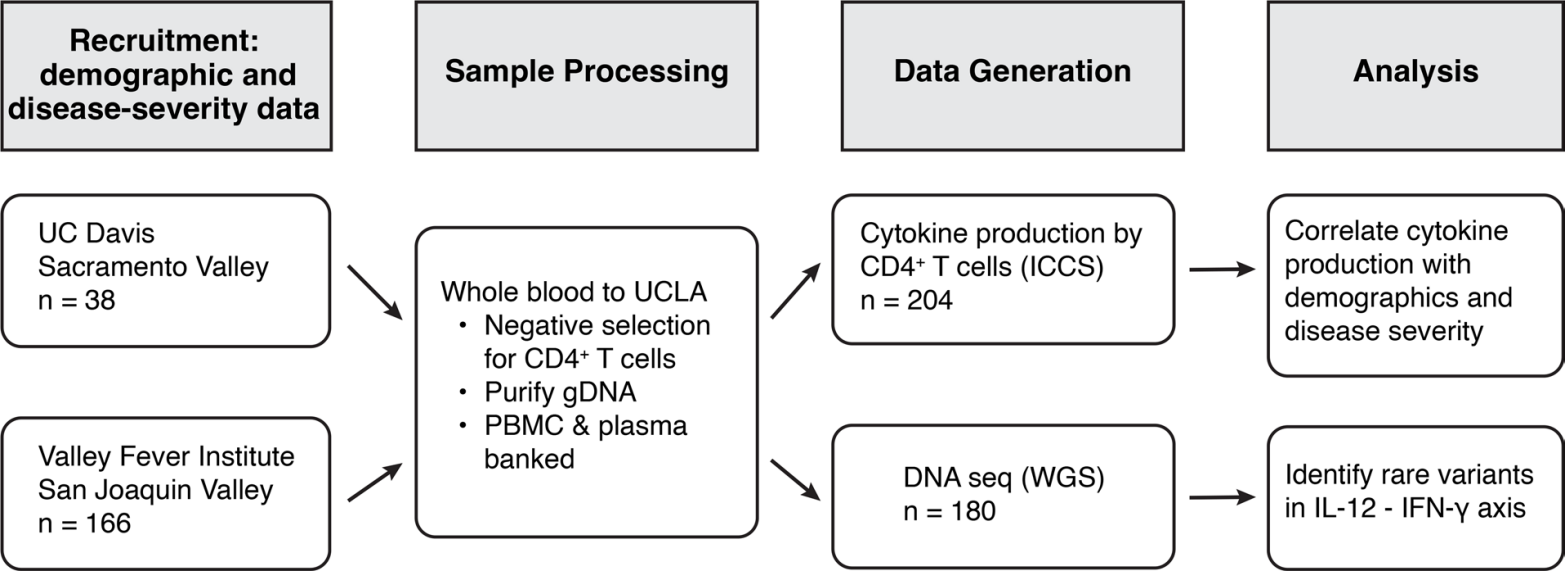
Flowchart demonstrating experimental design from recruitment to analysis.

Our patient cohort was drawn from the San Joaquin Valley of California and surrounding counties. They were recruited either at the Valley Fever Institute in Bakersfield or the Center for Valley Fever at the University of California, Davis. Our patient cohort was divided into the following clinical categories that we have previously described: 2, uncomplicated coccidioidomycosis; 3, complicated pulmonary disease (including patients with fibro-cavitary disease, persistent disease despite 6 months of treatment, and respiratory failure); 4, disseminated disease without meningitis; and 5, disseminated disease with meningitis (30). The relevant characteristics of our patient cohort are shown in Table 1. Of note, approximately two-thirds of individuals with complicated pulmonary and disseminated disease were male, and over half of our cohort self-identified as Hispanic or Latino. These demographics are in line with previous population-based studies on coccidioidomycosis disease burden in the central valley of California (31). The design of our study is shown in Figure 1.

**Figure 2:**
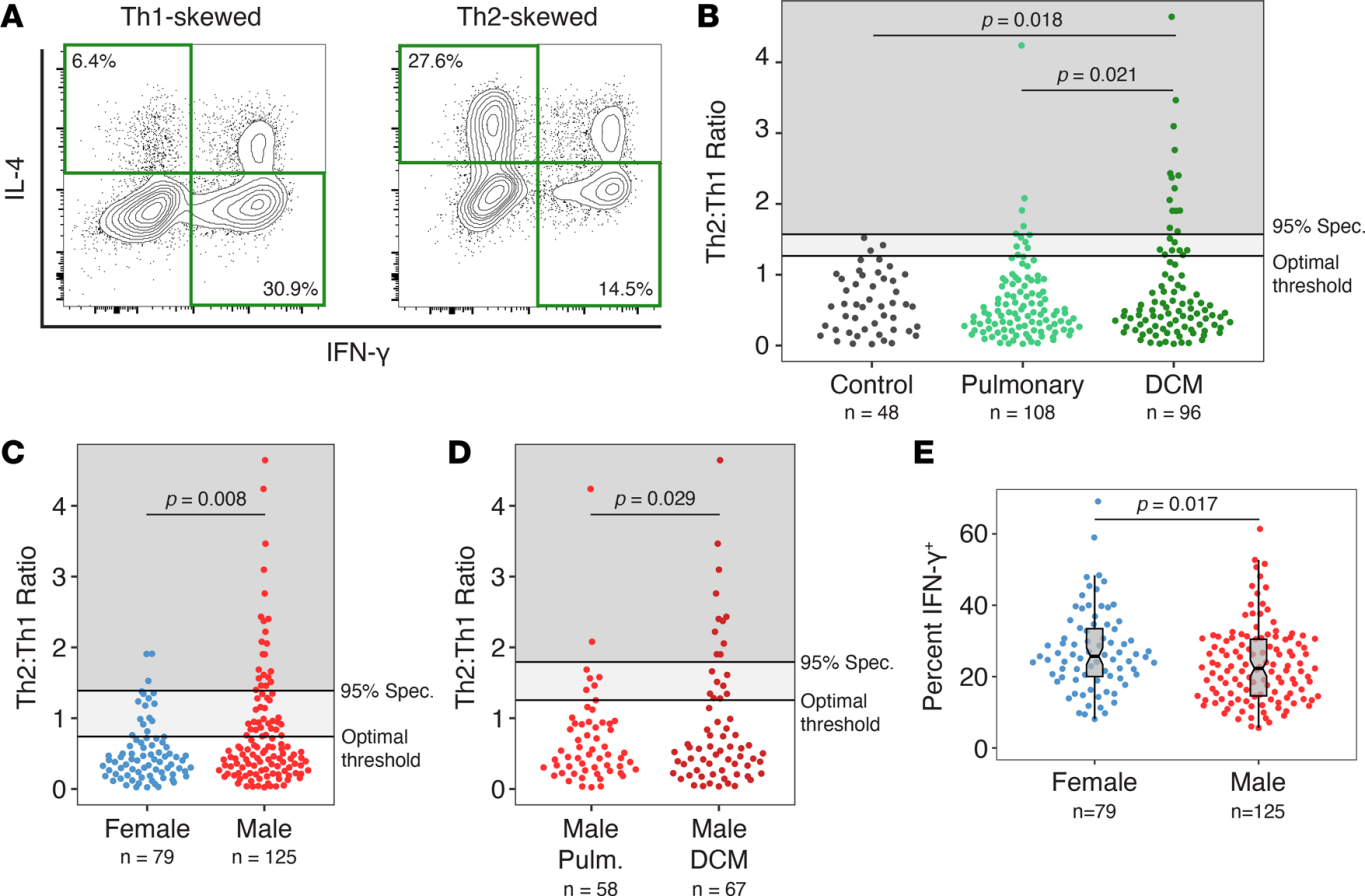
Th2 skewing of CD4^+^ T cells in coccidioidomycosis. (**A**) ICCS data for expression of IFN-γ and IL-4 was used to generate Th2/Th1 ratios. An example of a Th1- and Th2-skewed patient is shown. (**B**) Th2 skewing in controls and patients with pulmonary disease or DCM. Th2 skewing is significantly more common in patients with DCM (Fisher’s exact test at optimal cutoff; at 95% specificity, *P* = 0.005 (Cont. vs. DCM) and *P* = 0.017 (Pulm. vs. DCM). (**C**) Th2/Th1 ratio stratification by sex reveals that Th2-skewed individuals with DCM are predominantly male (Fisher’s exact test at optimal cutoff; at 95% specificity, *P* = 0.004). (**D**) Among males, significantly more patients had DCM than pulmonary-only disease (Fisher’s exact test at optimal cutoff; at 95% specificity, *P* = 0.020). (**E**) Females had more IFN-γ–producing cells than males (Wilcoxon’s rank-sum test). Box-and-whisker plots show the median (thick black bar in the gray box), interquartile range (top and bottom edges of gray box), and 95% CI (the narrowed waist of the gray box around the median). Each dot is 1 participant.

To measure cytokine production, we purified CD4^+^ T cells from both patients and healthy controls, activated them polyclonally, and performed intracellular cytokine staining (ICCS) on acutely restimulated cells. The proportions of Th1 cells (defined as IFN-γ^+^IL-4^–^) and Th2 cells (defined as IFN-γ^–^IL-4^+^) were determined (Figure 2A) and the Th2/Th1 ratio was calculated. Most controls and coccidioidomycosis patients had Th2/Th1 ratios less than 1, but among patients there was a marked tail of individuals with high Th2/Th1 ratios (Figure 2B and Supplemental Figure 1A; supplemental material available online with this article; https://doi.org/10.1172/jci.insight.199941DS1). To identify Th2-skewed patients, we calculated an optimal threshold (maximizing sensitivity and specificity) for the Th2/Th1 ratio using receiver operating characteristic (ROC) curves comparing pulmonary-only and disseminated patients (Th2/Th1 ratio ~1.27). We used the same ROC curves to set a second threshold at 95% specificity (Th2/Th1 ratio ~1.57). The proportion of coccidioidomycosis patients with a Th2/Th1 ratio greater than the optimal threshold increased with increasing disease severity, with significantly more DCM patients with a Th2-skewed phenotype than controls (OR 3.3, 95% CI [1.2, 12.8]; OR 8.1, 95% CI [1.0, 292.2] for 95% specificity threshold) or patients with pulmonary-only disease (OR 2.4, 95% CI [1.2, 5.5]; OR 2.9, 95% CI [1.2, 9.2] at 95% specificity) (Figure 2B). Together, these results reveal that a substantial fraction (23%) of patients with severe coccidioidomycosis showed Th2 skewing.

There are conflicting data on whether there is an inherent difference in Th1/Th2 bias in males and females (32, 33), with some studies showing enhanced Th1 skewing in males (34) and others in females (35). Given that male sex is a known risk factor for progression to DCM (36), we stratified our patient cohort by sex and examined the Th2/Th1 ratio, using thresholds generated from ROC curves comparing these groups. We found a strong bias toward male sex in Th2-skewed patients overall (OR 2.3, 95% CI [1.3, 4.6]; OR, 95% CI 4.0 [1.5, 15.2] at 95% specificity) (Figure 2C) and within the DCM group (OR 2.8, 95% CI [1.2, 7.9]; OR 4.0, 95% CI [1.0, 31.9] at 95% specificity) (Supplemental Figure 1B). Males with DCM were significantly more likely to be Th2 skewed than males with pulmonary-only disease (OR 2.5, 95% CI [1.1, 7.0]; OR 3.6, 95% CI [1.1, 19.0] at 95% specificity) (Figure 2D), while there was no difference for females in Th2 skewing between patients with DCM or pulmonary-only disease (Supplemental Figure 1C). In accord with the results for the Th2/Th1 ratio, females on average made significantly more IFN-γ than males (Figure 2E). Together, these results demonstrated an unexpected and marked sex bias of Th2 skewing in patients with severe coccidioidomycosis.

Some studies have shown that age may affect Th1/Th2 balance, with older adults biased toward Th2 responses, although contradictory results also exist (37–39). When we stratified our patient cohort by age and examined cytokine production, there was no discernible pattern. Th2-skewed patients were found in every age cohort (Supplemental Figure 1D). These results show no relationship between Th2 skewing and age.

Patterns of chemokine receptor expression on memory CD4^+^ T cells have been used as surrogate markers for helper T cell polarization (40). We measured chemokine receptor expression on CD4^+^ memory T cells from our patient cohort to determine whether these surrogate markers could be used to discern individuals who showed Th2 skewing (Supplemental Figure 2A). We compared the percentage of CD4^+^CD45RO^+^ memory cells with a CCR6^–^CXCR3^+^CCR4^–^ (Th1) or CCR6^–^CXCR3^–^CCR4^+^ (Th2) phenotype against the measured Th2/Th1 ratio of cytokine-producing cells (Supplemental Figure 2, B and C). The relationship between chemokine receptor expression and cytokine production showed the expected association (i.e., a negative slope when the percentage of cells with a Th1 chemokine receptor phenotype is plotted against Th2/Th1 ratio). However, only the correlation of Th2 memory cell levels with Th2/Th1 ratio reached statistical significance, and the contribution of percentage Th2 memory cells to the variance in Th2/Th1 ratio was modest (*r*^2^ = 0.12). These results suggest that the chemokine receptor expression on CD4^+^ T cells is not a good surrogate for cytokine production in these individuals.

Type-2 skewing of immune responses is found in atopic disease, in many cases characterized by eosinophilia and elevated levels of serum IgE (41). However, there were substantial numbers of atopic patients in all clinical categories (28%–41%), with no significant differences between clinical categories or between Th1-skewed (34.5%) and Th2-skewed (24.2%) patients (Table 1). We sought to determine whether patients with Th2 skewing in our coccidioidomycosis cohort had other hallmarks of severe atopic disease by comparing eosinophil counts and total serum IgE with the Th2/Th1 ratio, but no clear trends were present (Supplemental Figure 3, A and B). We note that not all patients had laboratory findings available, and only a handful of patients in our entire cohort had either eosinophilia or elevated IgE. Additionally, there was no correlation between eosinophil counts and IgE levels among patients for whom both measurements were available (Supplemental Figure 3C).

We also measured the plasma concentration of 9 cytokines associated with atopy in cohorts of Th2-skewed patients and age, sex, and disease-severity-matched Th1-skewed patients (Supplemental Figure 4A). While several atopic individuals showed high levels of the canonical Type-2 cytokines (IL-4, IL-5, IL-13, and IL-33), there was no correlation between plasma cytokine levels and Th skewing (Supplemental Figure 4B). Together, these results show that the presence of atopic disease was not predictive of Th2 skewing in our coccidioidomycosis cohort.

**Figure 3:**
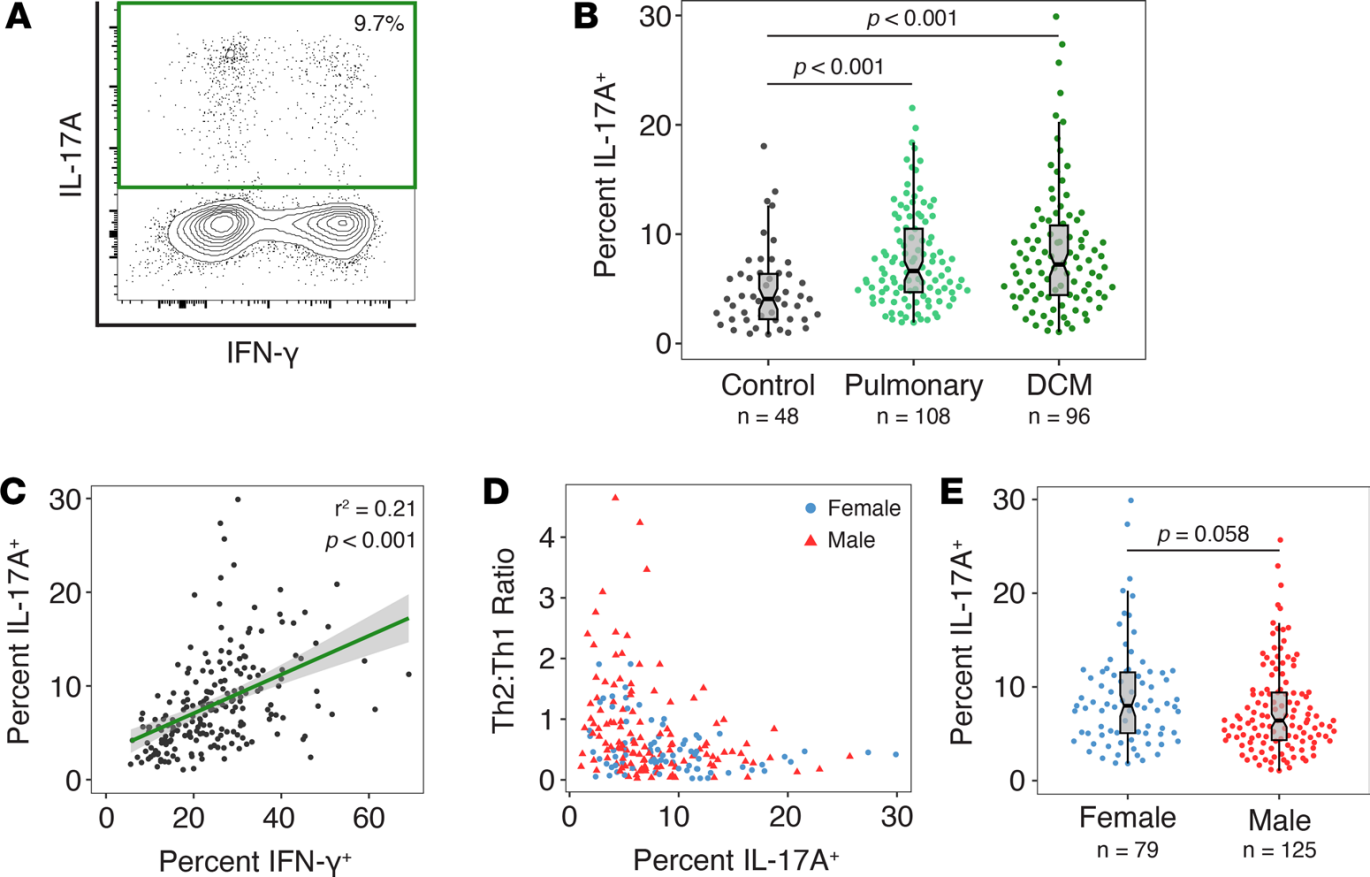
Analysis of Th17 polarization in patients with coccidioidomycosis. (**A**) Example of ICCS for measurement of Th17 polarization. (**B**) Coccidioidomycosis patients have significantly more Th17 cells than healthy controls, but no differences are seen when patients are stratified by disease severity (Kruskal-Wallis rank-sum test with Dunn’s post hoc test and Bonferroni’s correction). (**C**) Percentages of IL-17A– and IFN-γ–producing cells are correlated in coccidioidomycosis patients (Spearman’s rank correlation test). (**D**) Scatter plot showing an inverse correlation between percentage IL-17A–producing cells and Th2/Th1 ratio. (**E**) Male patients trended toward having fewer IL-17A–producing cells than females (Wilcoxon’s rank-sum test). Box plots show the median, interquartile range, and 95% CI (notch). Each dot is 1 participant.

The production of IL-17 cytokines by Th17 cells is crucial for mucosal defenses to fungal and bacterial infections. Both vaccine studies in mice (42, 43) and ex vivo analysis of T cells from pediatric patients (44) suggest that Th17 cells offer protection from coccidioidomycosis. Crucially, there is evidence that Th17 cells are important for preventing progression to DCM (4). Thus, we examined IL-17A production by CD4^+^ T cells (Figure 3A). Compared with healthy controls, we found a higher frequency of IL-17A–producing cells in patients with coccidioidomycosis. However, we did not observe any differences in Th17 skewing between pulmonary-only and DCM patients (Figure 3B). The percentages of IL-17A–and IFN-γ–producing cells were correlated within our coccidioidomycosis patients (Figure 3C). These results were highlighted by the fact that high Th2/Th1 ratios and large proportions of IL-17A–producing cells were mutually exclusive in our patient cohort (Figure 3D). When patients were segregated by sex, we found a trend toward a lower frequency of IL-17A–producing cells in male patients (*P* = 0.058) (Figure 3E), suggesting that Th2 skewing may come at the expense of Th17 defenses in some male individuals. Taken together, these results show that while IL-17A production does not vary by disease severity, Th2-skewed patients – predominantly males – have low proportions of Th17 cells.

**Figure 4:**
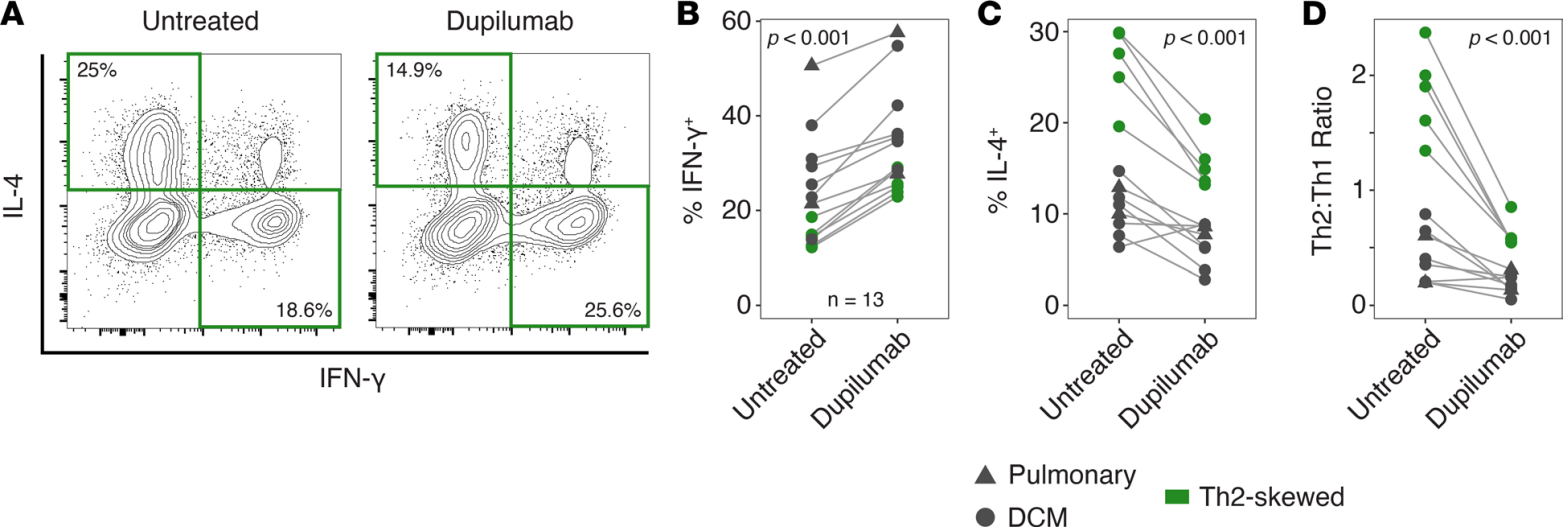
Dupilumab promotes IFN-γ production in individuals with Th2 skewing. (**A**) Representative examples of cytokine production by CD4^+^ T cells cultured in vitro with or without dupilumab. (**B**–**D**) Effect of dupilumab treatment on percentage IFN-γ^+^ cells (**B**), percentage IL-4^+^ cells (**C**), or Th2/Th1 ratio (**D**) (Wilcoxon’s signed-rank exact test).

We have previously demonstrated the efficacy of treating patients with refractory DCM and Th2 skewing with IFN-γ and dupilumab (IL-4R blockade) (27, 28). To demonstrate the efficacy of dupilumab alone in shifting the balance of cytokine production by CD4^+^ T cells ex vivo in patients with coccidioidomycosis, we cultured CD4^+^ T cells from patients in our cohort with or without dupilumab and measured cytokine production by ICCS (Figure 4A). Five of these patients showed Th2 skewing (Th2/Th1 ratio ≥ 1.27). In the presence of dupilumab, T cell cultures from all patients showed an increase in the percentage of IFN-γ–producing cells, and 12 out of 13 showed a decrease in both IL-4–producing cells and the Th2/Th1 ratio (Figure 4, B–D). This ex vivo result supports the notion that dupilumab may be able to remodel *Coccidioides*-specific CD4^+^ T cells in patients.

**Figure 5:**
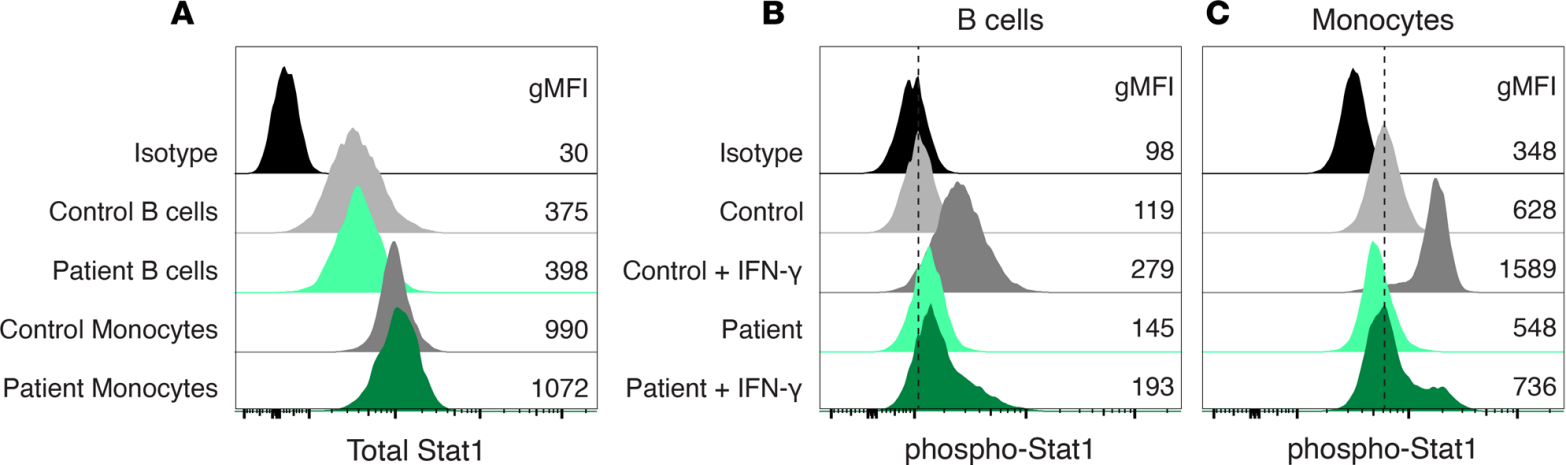
Validation of a rare variant in *IFNGR1* in a Th2-skewed patient with DCM. A heterozygous P431L variant in *IFNGR1* in a Th2-skewed patient with coccidioidomycosis (“Patient”) results in a hypomorphic response to IFN-γ. (**A**) Total Stat1 levels in control and patient B cells and monocytes. (**B** and **C**) Measurement of phosphorylation of Stat1 in response to IFN-γ stimulation shows a defective response in patient B cells (**B**) and monocytes (**C**). The geometric mean fluorescence intensities for all peaks are shown.

Patients with defects in Type-1 immunity are known to be susceptible to severe coccidioidomycosis (6). To determine whether deleterious alleles in the IL-12/IFN-γ axis were enriched in our cohort of Th2-skewed patients, we employed whole-genome sequencing (WGS). Both genomic data and cytokine profiling were collected for 180 patients. For this analysis, we studied those with a Th2/Th1 ratio of 1.0 or greater to avoid missing interesting edge cases. Among the DCM patients with a Th2 phenotype, we found 6 rare, interesting missense variants (Table 2). One, *IL12RB1* G447A, occurs with an allele frequency of 3.7 × 10^–6^ in gnomAD v4 and is predicted to be pathogenic by CADD, SIFT, PolyPhen, and ESM1b. Another, *IFNGR1* P431L, occurs with an allele frequency of 3.8 × 10^–5^ and is listed as a variant of uncertain significance in a ClinVar entry for disseminated atypical mycobacterial infection. We tested both rare variants in functional assays of cytokine signaling. Both B cells and monocytes from the patient with a P431L variant in *IFNGR1* had normal levels of Stat1 protein (Figure 5A) but showed a hypomorphic response in the phosphorylation of Stat1 upon stimulation with IFN-γ (Figure 5, B and C). Cells from the patient with a G447A variant in *IL12RB1* responded normally to IL-12 stimulation, as measured by phosphorylation of Stat4 (Supplemental Figure 5). These results demonstrate the feasibility of identifying rare variants in signaling pathways critical for Type-1 immunity in patients with Th2-skewed CD4^+^ T cells.

## Discussion

This work sheds light on how dysregulated immune responses may lead to severe coccidioidomycosis. We have demonstrated 3 important findings: (a) Th2 skewing of CD4^+^ T cells is present in a substantial fraction (~25%) of patients with DCM. This phenotype is correlated with disease severity; significantly more Th2-skewed individuals are found among those with disseminated disease. (b) Males are much more likely to have Th2-skewed T cells than females, and Th2 skewing in these individuals is concomitant with a decrease in Th17 cells. These results are concordant with the long-standing observation that males are more susceptible to severe coccidioidomycosis. However, most male patients with disseminated disease were not Th2-skewed. Thus, the overrepresentation of males among patients with DCM is multifactorial and may include non-biological factors (e.g., overrepresentation of males with occupational exposures, including construction and farm work). Nevertheless, our data suggest that screening male patients with severe disease for Th2 skewing will be fruitful, given the possibility of pharmaceutical interventions (IFN-γ and anti–IL-4R) for this population. (c) Genetic defects in the IL-12/IFN-γ signaling axis are known to increase susceptibility to mycobacterial infections (45) and have been implicated in several patients with severe coccidioidomycosis (6, 46). Here, we show that rare variants in the IL-12/IFN-γ axis exist in Th2-skewed patients with DCM. As a proof of principle, we measured signaling through the IFN-γ receptor in a patient harboring a heterozygous variant in the *IFNGR1* gene and showed a hypomorphic response.

One limitation of our study is the use of bulk CD4^+^ T cell cultures to measure cytokine production. Our rationale for using bulk cultures is 2-fold: (a) Cytokine production by bulk CD4^+^ T cells cultured under neutral conditions will necessarily be determined by a unique combination of genetics and epigenetic programming (determined by previous infections) that is reflective of an individual’s predisposition toward Type-1 or Type-2 T cell responses. (b) In a previous report, we showed that cytokine production in bulk T cells closely correlates with the T cell response to *Coccidioides* antigens ex vivo (27). Ideally, cytokine production by T cells specific for *Coccidioides* antigens would also be measured. Unfortunately, there are no existing coccidioides antigenic reagents that reproducibly activate T cells. For example, the T27 *Coccidioides* antigenic preparation (47, 48) stimulates robust upregulation of activation induced markers (CD69, CD134, and CD137) or cytokine production in fewer than half of clinically proven coccidioidomycosis patients. Our group and others (49) are currently developing *Coccidioides*-derived antigenic preparations to find reliable reagents for stimulating T cells in coccidioidomycosis patients with diverse HLA haplotypes.

These data showing a substantial number of Th2-skewed individuals among a large cohort of DCM patients are actionable clinically due to the demonstrated importance of Type-1 immunity in mediating a successful response to *Coccidioides* infection, and our ability to promote Th1-mediated immunity — at the expense of Th2-mediated immunity — by treating with IFN-γ and anti–IL-4R monoclonal antibodies (28). We suggest that patients with persistent *Coccidioides* infections, especially those with DCM and those not responding to antifungals — and especially males — should have their Th2/Th1 ratios tested ex vivo. We also suggest the actionability of screening patients found to have Th2 skewing for functionally relevant genetic defects in the IL-12/IFN-γ axis. Knowledge of such defects could inform treatment decisions and potentially improve patient outcomes.

## Methods

### Sex as a biological variable.

Patients were recruited without bias regarding biological sex. In the final cohort used in this report, there were 79 females and 125 males. The excess of males in our study is in line with previous reports demonstrating that severe coccidioidomycosis is more common in males (4). Healthy controls were anonymous, and neither their biological sex nor age is known.

### T cell culture.

CD4^+^ T cells were purified from fresh or frozen PBMCs by EasySep negative selection (STEMCELL Technologies, 17952). T cell blasts were generated by plating approximately 1 × 10^6^ purified CD4^+^ cells on 12-well plates coated with 1 μg/mL anti-CD3 (clone OKT3, BioLegend) in complete T cell medium (RPMI 1640 supplemented with 10% FCS, 10 mM HEPES, 1 mM sodium pyruvate, 55 μM 2-mercaptoethanol, and 1× Pen-Strep) with 2 μg/mL soluble anti-CD28 (clone CD28.2, BioLegend). On day 2 after stimulation, cells were replated in fresh medium supplemented with 50 U/mL IL-2. Cytokine production was measured on day 7–8. In some experiments, 10 μg/mL anti–IL-4R monoclonal antibody (generously provided by the Pediatric Allergy clinic at UCLA) (dupilumab) was included in the T cell cultures for the entire duration.

### ICCS.

Approximately 1 × 10^6^ CD4^+^ T cells were acutely stimulated with 50 ng/mL PMA and 1 μM ionomycin for 5 hours and 1× GolgiPlug (BD, 555029) was added for the final 4 hours of the stimulation. Cells were then fixed with PBS/2% paraformaldehyde and permeabilized with 1× Perm Buffer (BioLegend, 421002). Cells were stained in Perm Buffer with anti–IFN-γ (BioLegend, clone 4S.B3), anti–IL-4 (BioLegend, clone MP4-25D2), and anti–IL-17A (BioLegend, clone BL168). Data were collected on a Cytek DxP10 digital flow cytometer and analyzed with FlowJo software (Waters Biosciences).

### Phospho-Stat assay.

Thawed PBMCs were stimulated with 10 ng/mL rhIFN-γ (PeproTech, 300-02) or rhIL-12 (PeproTech, 200-12) for 20 minutes at 37°C. Stimulation was stopped by adding an equal volume of prewarmed Cytofix buffer (BD, 554655) and incubating for 12 minutes at 37°C. Fc receptors were blocked (TruStain FcX, BioLegend) for 5 minutes at room temperature, followed by a 20-minute stain on ice for CD19 (clone HIB19, BioLegend) and CD14 (clone HCD14, BioLegend) or CD4 (clone RPA-T4, BioLegend). Cells were permeabilized for 30 minutes on ice with 1 mL prechilled Phosflow Perm Buffer III (BD, 558050). After permeabilization, the cells were stained with anti–p-Stat1 pY701 (clone 4a, BD), anti–p-Stat4 p693 (clone 38/p-Stat4, BD), or isotype control (MOPC-173, BD) for 25 minutes at room temperature. The same staining procedure was used to measure total Stat1 levels in unstimulated samples with anti–Total Stat1 (clone 1/Stat1, BD) or isotype control (MOPC-173, BD). Data were collected on a Cytek DxP10 flow cytometer and analyzed with FlowJo software.

### Measurement of plasma cytokines.

Cytokines (IL-4, IL-5, IL-9, IL-13, IL-33, CCL11, Periostin, sRAGE, and TSLP) were measured by flow cytometry in thawed plasma samples with a bead-based multiplex assay, following the manufacturer’s instructions (LEGENDplex Allergy/Asthma Panel, BioLegend, 741472). Standard curves were generated with the ‘drc’ package in R (https://cran.r-project.org/bin/macosx/), using ‘LL.5’ within the ‘drm’ function to generate 5-parameter logistic models. The ‘ED’ function was used to interpolate cytokine concentrations from gMFI values. Data were collected on a Cytek DxP10 flow cytometer and analyzed with FlowJo software.

### DNA sequencing and quality control.

We conducted WGS on whole blood for 186 patients with coccidioidomycosis from our cohort. For the first batch of 88 samples, DNA was isolated using a Qiagen blood extraction kit, prepared with an Illumina TruSeq DNA PCR-free library kit, and sequenced using an Illumina NovaSeq 6000. The DRAGEN Germline Pipeline v3.2.8 (https://support.illumina.com/downloads.html) was used to align and map reads to the hg38-alt-aware reference on Illumina BaseSpace. Small variant joint calling was carried out with the DRAGEN Joint Genotyping Pipeline v3.7.5. The remaining participants were sequenced with Illumina Novaseq X Plus and processed using a similar DRAGEN pipeline on Amazon Web Services (AWS) designed by the UCLA SwabSeq lab.

Individual gVCFs were merged and indexed by chromosome using bcftools (7, 8). Variant INFO field annotations were recalculated in the merged files with the bcftools+fill-tags plug-in and alternate alleles not appearing in any sample were removed to convert to the final merged VCFs. The chromosome-level VCFs were then merged. The final merged VCF underwent quality control (QC) using bcftools (50, 51). The genotype for any sample that did not have a quality score of more than 20 or total read depth of more than 10 was set to missing. Duplicate variants were removed. We then converted the VCF to PLINK format and standardized variant naming. We filtered out variants with missing genotypes for more than 10% of samples, variants with a Hardy-Weinberg equilibrium *P* value of less than 0.0001, and samples with more than 10% of variants with missing genotypes (52–54). We used PLINK 2 to calculate KING-robust estimates of sample relatedness and removed one of each pair of individuals with a kinship coefficient of greater than 0.177 to remove first-degree relationships (55, 56). We also confirmed that the genetic sex of each patient matched the reported sex in our records. After QC, we had sequencing data for 180 patients with calculated Th2/Th1 ratios.

### Risk gene variant annotation and pathogenicity prediction.

From merged VCF files, we filtered to include only those variants appearing in 1 of 9 genes involved in Type-1 differentiation and signaling: *IL12A*, *IL12B*, *IL12RB1*, *IL12RB2*, *IFNG*, *IFNGR1*, *IFNGR2*, *ISG15*, and *TYK2*. We then used the Ensembl Variant Effect Predictor (VEP) tool to predict the consequences of each variant and add pathogenicity annotations (57). We also assessed the potential pathogenicity of all missense variants using the ESM1b and AlphaMissense machine learning models (58, 59). In R, we identified which variants passed quality control and calculated their allele frequencies in the QC-passed samples by coccidioidomycosis disease severity and by Th2 skewing (skewed, not skewed). Focusing on the variants with protein-coding consequences in their Ensembl canonical transcripts, we filtered to include only those variants that had at least 1 carrier with Th2 status assessed. We selected rare variants (gnomAD allele frequency < 0.01) with a higher allele frequency in DCM patients than in patients without dissemination and in Th2-skewed patients. Finally, we manually selected variants to prioritize for functional validation based on CADD Phred score, ClinVar annotations, and missense severity scores.

### Statistics.

All statistical analyses were conducted in R. Th2 skewing was determined by generating ROC curves comparing the Th2/Th1 ratio between 2 groups of patients with the ‘pROC’ package in R and then using the curves to identify thresholds. Optimal thresholds maximizing sensitivity and specificity were determined using the coords command with ‘best.method = youden.’ Thresholds for 95% specificity were similarly determined using the coords command. When comparing categorical data, we used Fisher’s exact test. ORs were calculated with the package ‘epitools,’ with the function ‘oddsratio’ and ‘method = small.’ When comparing the means of groups, we used the Shapiro-Wilk test to determine if the data were normally distributed. For comparisons where data from at least 1 group was not normally distributed, we used non-parametric tests: the Kruskal-Wallis rank-sum test and Dunn’s post hoc test with Bonferroni’s correction for multiple comparisons, and the Wilcoxon rank-sum test with continuity correction for comparison of 2 groups. For plasma cytokine experiments, comparisons between groups were measured with the Wilcoxon rank-sum test with Benjamini-Hochberg FDR correction. For experiments with paired samples, we used the Wilcoxon signed-rank exact test. Linear regressions and *r*^2^ values were generated in R with the function ‘geom_smooth’ and ‘method = lm.’ Correlations between 2 variables were tested with Spearman’s rank correlation test. A *P* value of less than 0.05 was considered significant.

### Study approval.

All patients provided written informed consent to participate in protocols approved by the Institutional Review Board (IRB) of UCLA, with reliance agreements from the Valley Fever Institute and the University of California, Davis.

### Patient recruitment.

All patients were evaluated by an infectious disease specialist from the Valley Fever Institute or the University of California, Davis, and were confirmed to have coccidioidomycosis. These experts also reviewed each patient’s disease trajectory and clinical status to assign scores using a clinicopathological categorization system that we have previously published (30). If the atopic status of patients was unknown, it was asked at their research visit. Patients were enrolled in an IRB-approved protocol, under which whole blood was collected and shipped to UCLA for analysis.

### Data availability.

Values for all data points in graphs are reported in the Supporting Data Values file. Genomic sequencing data are available in dbGap, accession number phs004536.v1.p1.

## Author contributions

Conceptualization: TJT, MIGL, and MJB. Investigation: TJT, SSN, AVS, AS, TSA, MAML, SLJ, CB, and MTT. Visualization: TJT, AVS, and SLJ. Funding acquisition: MJB. Supervision and project administration: MJB, MIGL, and VAA. Resources: GRT and RHJ. Writing – original draft: TJT. Writing – reviewing and editing: all authors.

## Conflict of interest

The authors have declared that no conflict of interest exists.

## Funding support

This work is the result of NIH funding, in whole or in part, and is subject to the NIH Public Access Policy. Through acceptance of this federal funding, the NIH has been given a right to make the work publicly available in PubMed Central.

- National Institute of Allergy and Infectious Diseases/NIH grants R21 AI149654 and U19 AI166059.
- University of California Office of the President grant VFR-19-633386.

## Supplementary Material

Supplemental data

ICMJE disclosure forms

Supporting data values

## Figures and Tables

**Table 1:**
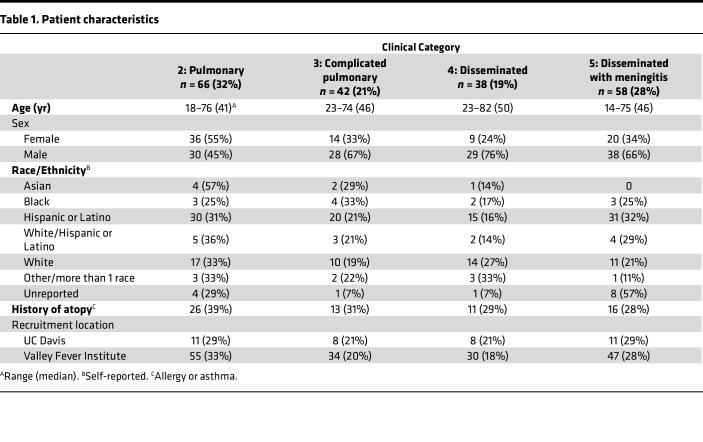
Patient characteristics

**Table 2:**
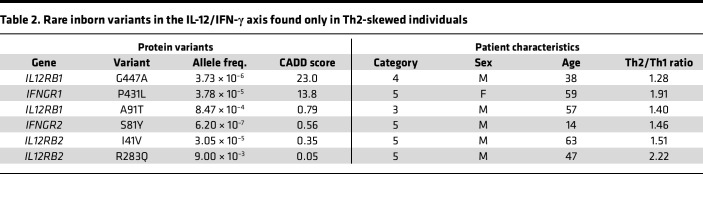
Rare inborn variants in the IL-12/IFN-γ axis found only in Th2-skewed individuals

## Version 1 04/21/2026

In-Press Preview

## Version 2 05/22/2026

Electronic publication
