# Supplementary material for: Th2 skewing in patients with disseminated coccidioidomycosis
Source: JCI Insight. 2026 Apr 21;11(10):e199941. doi: 10.1172/jci.insight.199941 (PMC13232725; doi:10.1172/jci.insight.199941)
Supplement: Supplemental data [file jciinsight-11-199941-s066.pdf]

Supplemental Figures

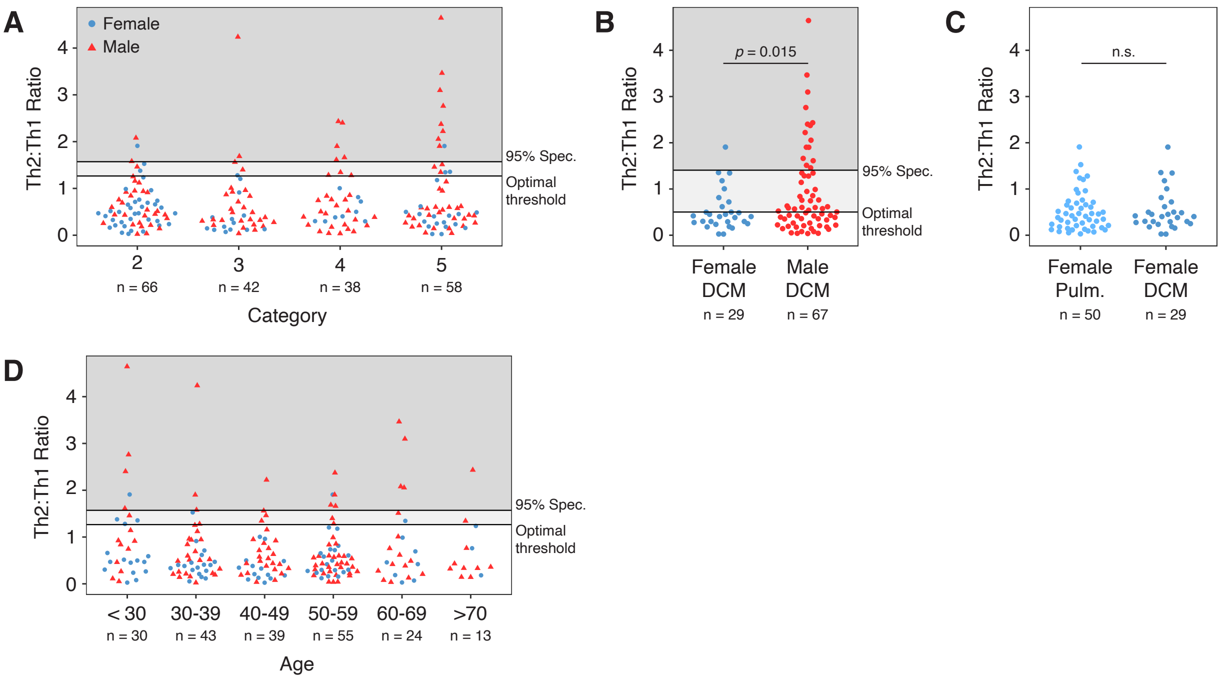

Supplemental Figure 1. (A) Distribution of Th2 skewed individuals across the four clinical categories. **(B)** Significantly more Th2-skewed males than females among DCM patients (Fisher's exact test at optimal cutoff; at 95% specificity, p = 0.034). **(C)** Females with pulmonary-only or DCM coccidioidomycosis show no difference in Th2 skewing. **(D)** Th2 skewing in coccidioidomycosis patients is not correlated with age.

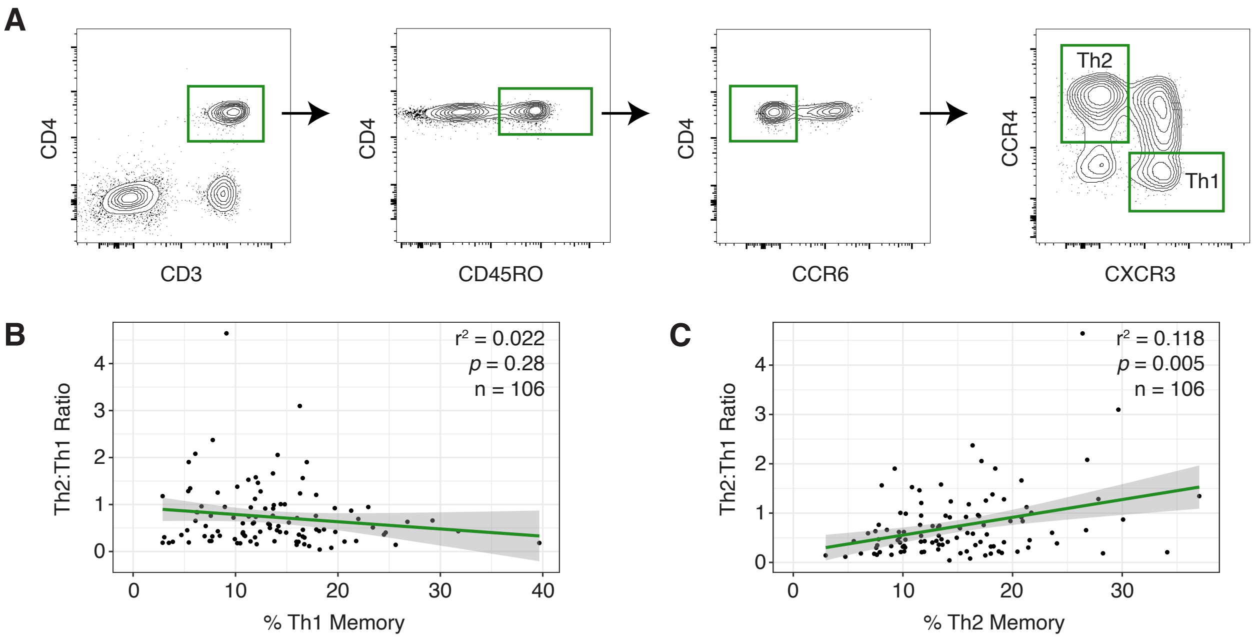

Supplemental Figure 2. (A) Gating scheme for identifying memory Th1 and Th2 cells. **(B and C)** Linear regressions with 95% CI between the Th2:Th1 ratio and the percent Th1 (CCR6$^-$CXCR3$^+$CCR4$^-$) **(B)** or Th2 (CCR6$^-$CXCR3$^-$CCR4+) **(C)** cells among CD4$^+$CD45RO$^+$ memory T cells in coccidioidomycosis patients (Spearman's rank correlation test).

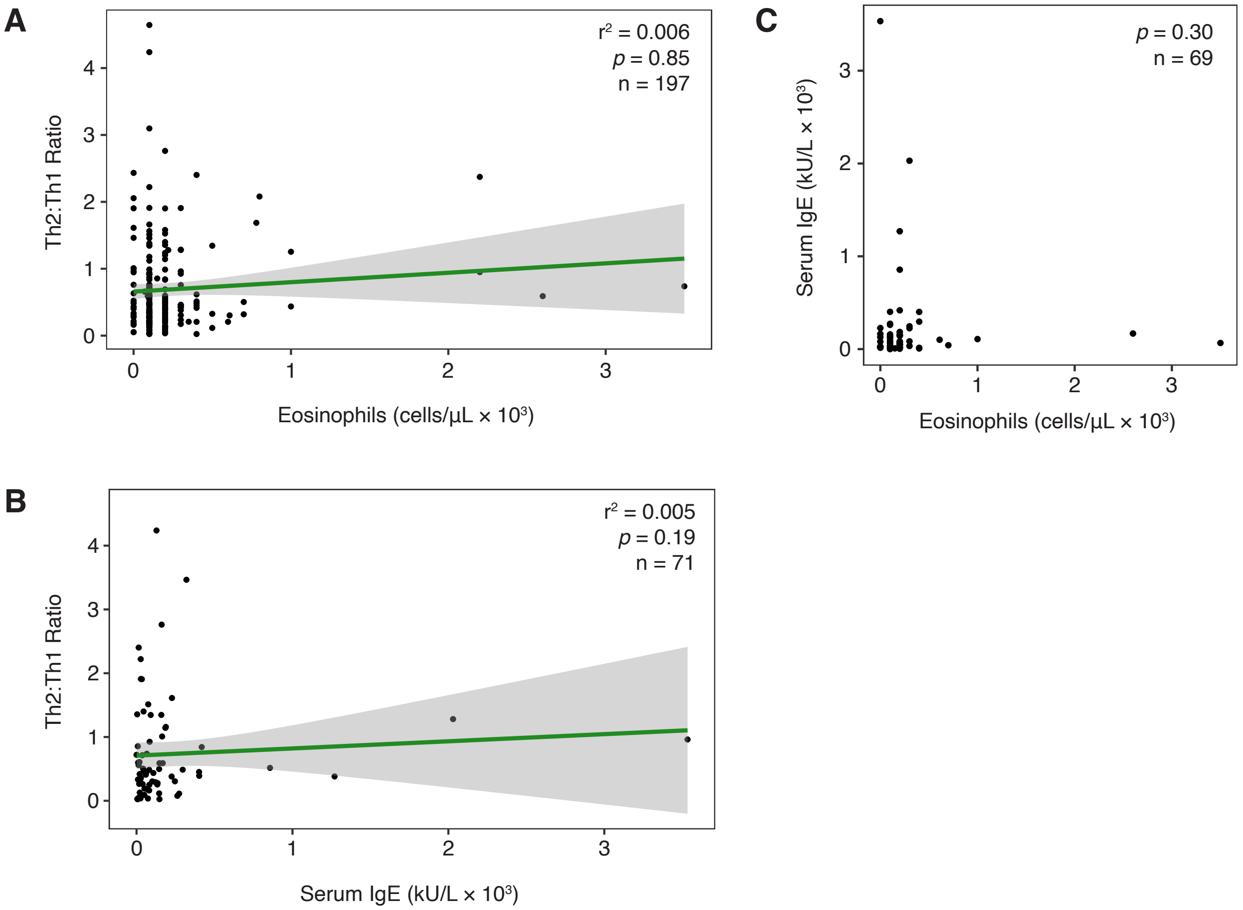

Supplemental Figure 3. (A and B) Linear regressions with 95% CI between the Th2:Th1 ratio and absolute eosinophil counts (A) or serum IgE (kU/L) (B) (Spearman's rank correlation test). **(C)** Absolute eosinophil counts are plotted vs serum IgE, showing the absence of a relationship in our dataset.

A

	Age*	Sex	Pulmonary	DCM
Th1 skewed	48 (50)	13M, 2F	6	9
Th2 skewed	48 (51)	12M, 3F	6	9

*Mean (median)

B

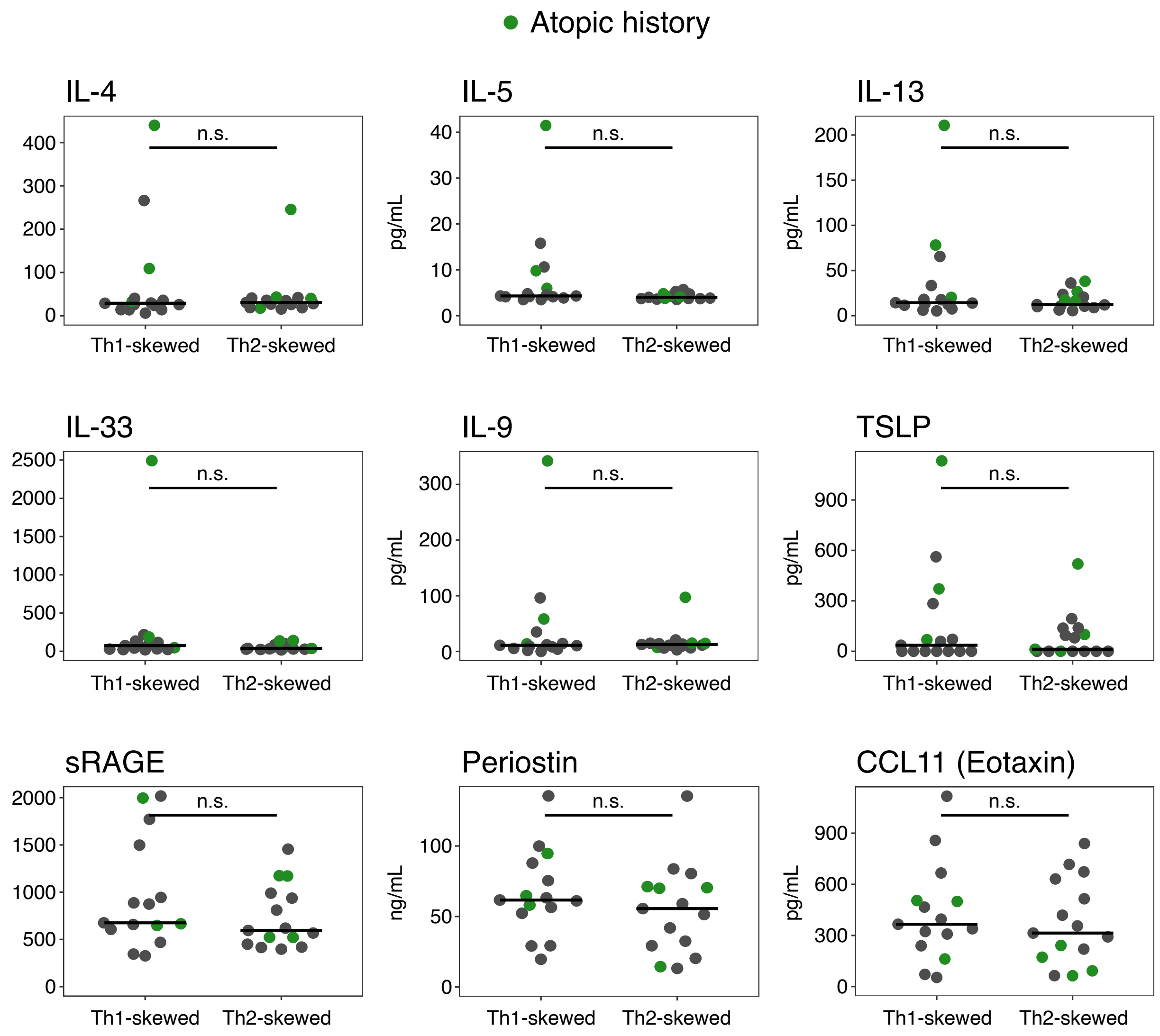

Supplemental Figure 4. (A) Characteristics of patients whose plasma was assayed for Type-2 cytokines. **(B)** Plasma cytokines were measured by a bead-based flow cytometry assay. There were no significant differences between Th1 and Th2 skewed patients (Wilcoxon rank sum test with Benjamini–Hochberg FDR correction). Medians are shown.

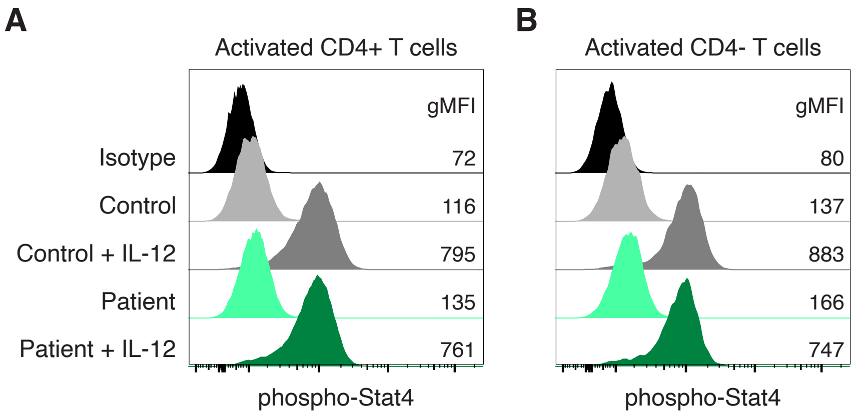

Supplemental Figure 5. (A and B) Measurement of phosphorylation of Stat4 in response to IL-12 stimulation shows a normal response in $CD4^{+}$ **(A)** and $CD4^{-}$ **(B)** activated T cells from a Th2-skewed patient with a G447A mutation in *IL12RB1*. The geometric mean fluorescence intensities for all peaks are shown.
